# Adrenergic effect on women’s blood neutrophil oxidative activity the first day after delivery

**DOI:** 10.5935/1518-0557.20240025

**Published:** 2024

**Authors:** Oksana Olegovna Zaitseva, Victor Ivanovich Tsirkin, Inna Gennadievna Paturova, Tatiana Vitalievna Polezhaeva, Olga Nurzadinovna Solomina, Andrey Nikolaevich Khudyakov, Marta Igorevna Sergushkina, Svetlana Leonidovna Dmitrieva

**Affiliations:** 1 Institute of Physiology of Komi Science Centre of the Ural Branch of the Russian Academy of Sciences, FRC Komi SC UB RAS, Syktyvkar, Komi Republic, Russia; 2 Kazan State Medical University, Kazan, Republic of Tatarstan, Russia; 3 Kirov State Medical University, Kirov, Kirov region, Russia; 4 Kirov Regional Clinical Perinatal Center, Kirov, Kirov region, Russia

**Keywords:** pregnancy, childbirth, neutrophils, radical activity, adrenergic receptors.

## Abstract

**Objective:**

Recent studies have described a significant role for neutrophils in reproductive processes and their participation in the preparation of the cervix for childbirth and the activation of labor, in the postpartum involution of the uterus, and in the occurrence of preeclampsia. This study aimed to assess the formation of free radicals by neutrophils in the blood of women on the first day after childbirth and to characterize the adrenergic effect on this process.

**Methods:**

Venous blood samples from 100 female volunteers aged 26-32 years who had 2 or 3 full-term deliveries were collected and analyzed. Various adrenergic compounds were considered (agonists alphaand betaadrenoreceptors, adrenoblockers). The intensity of the respiratory burst of neutrophils and the effect of adrenergic substances on them were assessed with latex-induced luminol-dependent chemiluminescence.

**Results:**

Neutrophil activity depends on the stage of the woman’s reproductive process: it decreases during pregnancy, reaches the lowest values during childbirth, and increases significantly in the first hours after childbirth. On the first day after childbirth, alpha-1-adrenergic receptors are highly active in neutrophils, through which NADP-H-oxidase is activated and activated oxygen species are formed. At the same time, alphaor beta-agonists inhibit the radical activity of cells.

**Conclusions:**

Latex-induced oxidative burst of female blood neutrophils correlates with the stage of the reproductive process. Stressful conditions in the postpartum period can suppress the ability of neutrophils to release reactive oxygen species, which increases the risk of postpartum infections.

## INTRODUCTION

In recent years, our understanding of the physiological significance of neutrophils in pregnancy has substantially increased (Tsirkin *et al*., 2015). Some authors believe they can induce birth, both term and preterm, and take part in the uterus involution process (Shynlova *et al*., 2013) and in the development of pre-eclampsia (Lampé *et al*., 2015). It is believed that during pregnancy uterine contractile activity is regulated by many mechanisms, one of which is beta-adrenoreceptor inhibitory mechanism (beta-ARIM) (Tsirkin *et al*., 2014). The myometrium contains all types of adrenoceptors. Regulation of their quantity and activity ratio determines the course of pregnancy. In particular, activation of beta-adrenergic receptors (beta-AR) of the uterus leads to a decrease in myocyte contractile activity, which is associated with increased pregnancy success (Tsirkin *et al*., 2014).

Epinephrine and norepinephrine play an important role in the regulation of both innate and acquired immunity. Catecholamines produce immunomodulatory effects by interacting with alpha-adrenergic receptors (alpha-AR) and beta-AR on various immune cells. Phagocytes can synthesize and release both catecholamines and adrenergic receptors (Flierl *et al*., 2007).

The literature shows that neutrophils contain both alpha-AR and beta-AR, which are further subdivided into different subtypes including alpha-1A, alpha-2C, alpha-1D, beta-1, beta-2 (Kanashiro *et al*., 2020). The presence of alpha-AR in neutrophils in various physiological conditions has been confirmed by numerous studies (Flierl *et al*., 2007; Herrera-García *et al*., 2014). It has been established that stimulation of alpha-1-AR results in increased neutrophil migration, while stimulation of alpha-2-AR produces an opposite effect (Kanashiro *et al*., 2019).

Stimulation of the G_q_ protein (alpha subunit is a family of heterotrimeric G protein alpha subunits) by alpha-1-AR leads to the activation of phospholipase C, which produces inositol triphosphate, an intracellular messenger triggering intracellular Ca2+ release, followed by increased neutrophil migration. Ca2+ also activates intracellular protein kinase C (PKC), which plays an essential role in the phosphorylation of the cytosolic NADPH-oxidase (nicotinamide adenine dinucleotide phosphate oxidase) subunit, p47phox (Fontayne *et al*., 2002). It should be noted that all NADPH-oxidase subunits (gp91-phox, p22-phox, p47-phox, p67-phox, p40-phox) are separated in a resting cell to prevent tissue injury (Quinn & Gauss, 2004). When activated by several stimuli, they unite and form an enzyme complex that generates and releases superoxide free radicals, which are the precursors of other reactive oxygen species (ROS) combating bacterial infection (Quinn & Gauss, 2004).

Studies have found that epinephrine and norepinephrine provide an anti-inflammatory effect by binding to and activating alpha-2-AR (Kanashiro *et al*., 2019). Stimulation of the G_i_ protein by alpha-2-AR results in the neutrophil migration inhibition _(_Herrera-García *et al*., 2014). Beta-2-AR desensitization can significantly reduce inflammation (Flierl *et al*., 2007). *In vitro* studies have shown that stimulation of beta-AR inhibits the capability of neutrophils to form extracellular traps (Marino *et al*., 2018), their oxidative burst and chemotaxis (Nielson, 1987; Barnett *et al*., 1997; Scanzano *et al*., 2015).

The inhibition of neutrophil oxidative burst capacity can be mediated either by cAMP-dependent (Nielson, 1987; Bazzoni *et al*., 1991; Gibson-Berry *et al*., 1993) or cAMP-independent (Brunskole Hummel *et al*., 2013) mechanisms. The presence of beta-3-AR in the neutrophil membrane is still being discussed (Berkowitz *et al*., 1995; Marino *et al*., 2018).

Due to a wide range of biological functions regulated by AR, it is reasonable to consider them as potential therapeutic targets for various diseases. To better understand the pathogenesis of obstetrical conditions, their diagnosis and treatment, it is important to determine the role of adrenergic compounds in the mechanisms of oxygen-derived free radical generation in neutrophils at different stages of the reproductive process.

In this regard, the purpose of this work is to assess the formation of free radicals by neutrophils in the blood of women on the first day after childbirth and to characterize the adrenergic effect on this process.

## MATERIAL AND METHODS

### Object of research

Heparinized venous blood samples of 35 female volunteers, aged 26-32 years, with two or three full term births, were collected and studied. Each woman had a blood sample taken once. A total of 235 tests were performed. Pregnancy occurred without any complications; labor occurred at 39-40.5 (40 weeks on average) weeks of gestation. This study was conducted in accordance with the principles established by the Helsinki Declaration (WMA, 1964), all subsequent amendments and also the ‘‘Law of the Russian Federation on the donation of blood and its components.’’ All patients provided written informed consent. All trials were approved by the hospital and the institutional review board of the Institute of Physiology of Komi Science Centre of the Ural Branch of the Russian Academy of Sciences.

Blood samples were collected from the median cubital vein with the aid of vacuum tubes (Ningbo Greetmed Medical Instruments Co., Ltd., China) containing Na-heparin, in the first 12 hours after childbirth in the obstetric unit of Kirov Region Perinatal Center (Kirov, Russia). The blood samples were analyzed within two hours of collection. The samples were transported to the laboratory at +4°C within 20 minutes of collection.

### Materials and chemicals

The following adrenergic compounds (at a final concentration of 1 µg/L) were selected for further analysis: alpha-1-AR agonist phenylephrine (Irifrin, Sentiss, Switzerland), beta-2-AR agonist hexoprenaline sulfate (Ginipral®, Nycomed, Austria), beta-3-AR agonist mirabegron (Betmiga®, Astellas Pharma Technologies Inc., USA), alpha-beta-AR agonist epinephrine (Adrenaline, Moscow Endocrine Plant, Russia), alpha-blocker nicergoline (Armavirskaya biofabrika, Russia), beta-blocker propranolol hydrochloride (Anapriline, ”Biosynthesis”, Russia), beta-1 blocker metoprolol (Merkle GmbH, Germany), beta-3 blocker SR-6 (Sigma). In addition, Hanks’ balanced salt solution (HBSS) (BioloT, Russia), luminol (FlukaBioChemika, Switzerland), and latex beads of 0.08 µm in diameter (Sigma-Aldrich, Germany) were used.

To prepare the latex working solution, 0.1mL of latex beads was diluted in 1mL of HBSS.

To prepare luminol working solution, 1mg of dry substance was dissolved in 750µL of 1 N potassium hydroxide and brought to 50mL with distilled water. It is worth mentioning that it is possible to determine total functional activity of neutrophils using luminal as an activator, since the latter can react with primary and secondary ROS (hydroxyl radical, hydrogen peroxide, etc.) in the intracellular space and outside the cell.

*The respiratory burst level* was evaluated using a BHL-07 biochemiluminometer (OOO Medozons, Russia). The following characteristics of the chemiluminescent reaction were identified: the maximum intensity value Imax (mV), which shows the maximum level of ROS production; and the area under the chemiluminescence curve S (mV×s), which integrally characterizes ROS generation for 30 min.

To assess spontaneous neutrophil activity, 0.1mL of blood was mixed with 0.05mL of latex solution. Then, 0.05mL of the mixture was put into a measuring cell where 0.95 mL of HBSS and 0.2mL of luminol solution were added. The measuring cell with the mixture was placed into the biochemiluminometer measuring chamber for 30min. Mixing and temperature control modes were switched on (+37°C).

### Assessment of the effect of adrenergic substances on the intensity of the neutrophil respiratory burst

To assess the effect of adrenergic substances on neutrophil radical activity, 0.05mL of solution of the substance at a concentration 1µg/L and 0.05 ml of latex solution were added to 0.1mL of blood. Then, 0.05mL of the mixture was placed into a measuring cell. Then, 0.95mL of Hanks solution and 0.2mL of luminol working solution were added. The measuring cell with the reaction mixture was placed into a biochemiluminometer measuring chamber for 30min. The mixing and temperature control modes were switched on (+37°C).

To assess the combined influence of an adrenergic agonist and an adrenergic blocker, 0.05mL of each substance solution (1µg/L) and 0.1 mL of latex solution were added to 0.1mL of blood. Then, 0.2mL of the mixture was placed into a measuring cell. Next, 0.8mL of Hanks solution and 0.2mL of luminol working solution were added. The measuring cell with the reaction mixture was placed into a biochemiluminometer measuring chamber for 30min. The mixing and temperature control modes were switched on (+37°C).

### Statistical analyses

The statistical analysis of the obtained data was performed on software package BioStat 2009 Professional 5.8.4 (АnalystSoft, USA). To assess the differences, the nonparametric Mann-Whitney-Wilcoxon test was used with a significance level of 0.05. The results of the study are presented in the tables and in the text as the median and the 25th and 75th percentiles (*Me, Q1-Q3*).

## RESULTS

### Neutrophil activity background level

The results of previous studies have shown that the latex-induced radical activity of neutrophils (LIRAN) depends on the stage of the woman’s reproductive process (Polezhaeva *et al*., 2020). It has been established that LIRAN statistically decreases in non-pregnant women in the follicular phase of the ovarian cycle (group 2), as well as during pregnancy, especially in the second and third trimesters (groups 4 and 5). It is important to note that LIRAN reaches the lowest levels during childbirth (decreases by 4.4 times in comparison with group 1). In this study, LIRAN reached its highest values in the first hours after childbirth (Table 1). The summarized data are presented in Table 1.

**Table 1:** Parameters of luminol-dependent latex-induced chemiluminescence of female blood neutrophils at different stages of reproduction.

Group of Women		Group Number	Chemiluminogram Parameters	
			S(мV×с)	I_мах_ (мV)
Non-pregnant Women, the luteal phase of the ovarian cycle		1	76945 (56370-110493)^*2-7^	96 (75.5-135.5)^*2-7^
Non-pregnant Women, the follicular phase of the ovarian cycle		2	23470 (19399-46378)	28 (25-44)
Pregnant Women having a successful healthy pregnancy	trimester I	3	50671 (31517-56984)	48 (33-68)
	trimester II	4	29837 (27473-37398)	34 (27-47)
	trimester III	5	38465 (10981-65681)	39 (19-77)
Parturient Women in the first stage of uncomplicated labor		6	17358 (10602-19870)^*1-5,7^	22 (15-24)^*1-5,7^
New mothers (the first day after labor)		7	109658 (67133-167237)^*2-6^	101 (60-143.5)^*2-6^

Note: Number of women in each study group - 10;

* -statistically significant differences with data from other groups *p*<0.05.

### Influence of adrenergic substances on the neutrophil respiratory burst

Against a background of high functional activity of neutrophils, the AR agonists used in this study, including mirabegron beta3-AR agonist, significantly reduced it (Table 2). Differences in the effect produced by the different AR agonists were not statistically significant. We also found that on the background of each of the adrenergic blockers used, epinephrine had a stimulating effect.

**Table 2:** Parameters of luminol-dependent latex-induced chemiluminescence of women’s blood neutrophils the first day after labor when exposed to adrenergic compounds at 1 µg/L concentration.

Substance	Group Number	Chemiluminogram Parameters	
		S (мV×с)	I_мах_ (мV)
Background value	1	181375.5 (114344.5 - 356679.3)	201.5 (118 - 319)
+ Hexoprenaline sulfate	2	61965 (43617.3 - 82903) ^*1^	58 (39.5 - 79.3) ^*1^
+ Phenylephrine	3	79133 (67748.5 - 142225.5) ^*1^	70 (52.8 - 113.3) ^*1^
+ Mirabegron	4	68132 (48711.5 - 148885) ^*1^	83 (42.5 - 133.5) ^*1^
+ Epinephrine	5	87230 (72221.3 - 143157.5) ^*1^	103.5 (64 - 128.8) ^*1^
+ SR-6 + epinephrine	6	418 044 (211472.8 - 757526.3) ^*5^	721.5 (312.5 - 1059.8) ^*5^
+ Metoprolol + epinephrine	7	325687 (167653.3 - 522419.3) ^*5^	427.5 (259.5 - 622.8) ^*5^
+ Propranolol hydrochloride + epinephrine	8	508986.5 (386771.8 - 960760.8) ^*5^	917.5 (520.5 - 1284.8) ^*5^
+ Nicergoline + epinephrine	9	349081 (322050 - 442433) ^*5^	640 (388 - 848) ^*5^

Notes: Number of studies in each group - 25;

* - statistically significant differences with data from other groups *p*<0.05.

A high background functional activity of neutrophils was established. However, all alphaor beta-AR agonists used in the study, including the beta3-AR agonist mirabegron, significantly suppressed the radical activity of cells (Table 2). Differences in the effect of AR agonists were not statistically significant. Moreover, we found that adrenaline can increase the secretion of ROS by neutrophils in puerperas during desensitization of alpha-AR or beta-AR/beta-1-AR/beta-3-AR.

## DISCUSSION

We have shown that in women of reproductive age, the capability of neutrophils to generate ROS in response to latex stimulation depends on the menstrual cycle phase. During the luteal phase of the menstrual cycle, the level of progesterone produced by the corpus luteum is significantly higher, leading to increased neutrophil oxidative burst activity. During the follicular phase, when progesterone levels are low and estradiol (estrogen) levels are high, neutrophil activity is reduced (Polezhaeva *et al.,* 2021). It is known that progesterone is essential for the regulation of female normal reproductive function. The physiological effects of progesterone are mediated by the interaction of the hormone with either nuclear progesterone receptors nPR-A, nPR-B and nPR-С (a genomic mechanism) or membrane progesterone receptors mPR-alpha, mPR-beta and mPR-gamma (a nongenomic mechanism) (Kowalik *et al*., 2013). The nuclear progesterone receptor has two main isoforms: A (PGRA) and B (PGRB). PGRB activates progesterone responsive genes, while PGRA is a weaker activator and can act as an inhibitor of PGRB activity. The ratio of the two isoforms changes during the estrous cycle and pregnancy, and correlates with the level of progesterone signaling, occurring in the reproductive tract. Membrane progesterone receptors rapidly activate appropriate intracellular signal transduction pathways, and, as a result, they can initiate specific cell responses (Kowalik *et al*., 2013). These observations suggest that high progesterone levels during the luteal phase of menstruation might be the cause of increased neutrophil oxidative burst activity. The latter is necessary to prepare a woman’s body for pregnancy. Increased oxidative burst activity of women’s neutrophils during the luteal phase is likely due to the activation of nPR-B and genes controlling the NADPH-oxidase subunits, i.e. gp91-phox, p22-phox, p47-phox, p67-phox, p40-phox. Decreased neutrophil activity during the follicular phase is probably due to an increasing level of estrogen, which reduces the ability of progesterone to increase the expression of NADPH-oxidase subunit genes.

The analysis of luminol-dependent latex-induced chemiluminescence parameters has shown that in healthy pregnancies as well as in cases of threatened miscarriage (with pregnancy maintenance), neutrophils are in an activated (or primed) state and equally responsive to latex induction. However, neutrophil deactivation occurs during labor. At the same time, the intensity of free radical formation in parturient women is the same as in non-pregnant women, which is explained by the migration of activated neutrophils into the myometrium (Polezhaeva *et al*., 2020). We believe that against a background of an increased level of progesterone, its ability to change the ratio of adrenoreceptors on neutrophil membranes through genomic pathways is manifested. It is possible that progesterone increases the expression of beta-AR and/or reduces alpha-1-AR, which ultimately reduces the production of ROS. We think that this decrease is linked to increased progesterone levels during pregnancy. Together with other substances, for example, acetylcholine (Zhang *et al*., 2020), progesterone facilitates the development of maternal immunological tolerance to a semiallogeneic fetus inhibiting the ability of dendritic cells, monocytes and macrophages, to present antigens to Th-cells (T helper cells, also known as CD4+ cells or CD4-positive cells) (Krzymowski & Stefańczyk-Krzymowska, 2012). Progesterone synthesis increases significantly, due to its production by the placenta as well. Progesterone is essential for the maintenance of pregnancy, as it regulates the expression of a large number of genes. It increases the expression of beta-AR, potassium channels in uterine myocytes and, thus, contributes to beta-ARIM and potassium myometrium-inhibitory mechanism formation (Tsirkin *et al*., 2014). Through the membrane receptors of the G-protein, progesterone inhibits adenylate cyclase and myosin light chain phosphorylation, thus reducing myometrium contractile activity (Karteris *et al*., 2006; Mesiano, 2007).

In the first stage of uncomplicated labor, the parameters of latex-induced chemiluminescence of neutrophils are significantly lower than those during pregnancy. We suppose that this decrease is due to several reasons. According to the literature, leukocytes actively migrate to the reproductive tract before childbirth, where they produce cytokines and initiate labor (Luppi *et al*., 2004; Yuan *et al*., 2009; Shynlova *et al*., 2013). However, the white blood cell count does not decrease. Therefore, it can be assumed that blood cells with the highest oxidative burst activity leave for the reproductive tract. Another reason for the decrease in neutrophil response may be the phosphorylation inhibition of p47phox, a key regulatory NADPH-oxidase cytosolic subunit. It should be clarified that neutrophil stimulation promotes the co-assembly of NADPH-oxidase subunits: the p47phox-p67phox-p40phox complex (cytosolic regulatory protein of the corresponding molecular weight 47-kDa, 67-kDa, 40-kDa of the NADPH-oxidase 2 complex) is formed in the cytoplasm; p47phox becomes phosphorylated to translocate to the membrane-bound complex (gp91phox and p22phox) (Babior, 1999; Quinn & Gauss, 2004; Sheppard *et al*., 2005). Neutrophil stimulation induces a change in the p47phox conformation that correlates with respiratory burst activity (Quinn & Gauss, 2004). Any of the four PKC isoforms - α, β, δ, and ζ - may participate in р47phox phosphorylation (Sheppard *et al*., 2005). We hypothesize that during childbirth p47phox phosphorylation is impaired due to PKC deactivation (possibly due to a deficiency of cytosolic Ca2+) in order to stop the oxidase reaction, which is insignificant for the body at the moment. Evidence suggests that the respiratory burst depends not only on phosphorylation (a positive effect of kinases), but on dephosphorylation of oxidase subunits under the influence of phosphatases (a negative effect of phosphatases) as well (Heyworth *et al*., 1997).

The present study revealed and confirmed that in the first 24 hours after childbirth, neutrophils showed a significant increase in ROS generation in response to latex stimulation, i.e. neutrophil free radical production significantly increased at levels higher than in the luteal phase of the menstrual cycle in non-pregnant women. This fact is of great importance from a physiological point of view. High oxidative activity of female blood neutrophils the first day after delivery aims to protect the mother from genital and extragenital infections, and also contributes to postpartum uterine involution. We suggest that during this period, alpha-1 adrenoceptor subtypes become prevalent and stimulate the neutrophil NADPH-oxidase activation.

The chemiluminograms obtained demonstrate that both alphaor beta-adrenergic agonists may inhibit the neutrophil respiratory burst. The physiological significance of such ROS response of neutrophils to catecholamines probably lies in the formation of a mechanism that prevents excessive activity of neutrophils in this period and implement with the participation alpha-2-AR and / or beta-2-AR. Other authors have confirmed this hypothesis (Nielson, 1987; Barnett *et al*., 1997; Herrera-García *et al*., 2014; Scanzano *et al*., 2015). Decreased oxidative burst in neutrophils also suggests that stressful conditions in the postpartum period can inhibit neutrophils’ capability to generate ROS, resulting in an increased risk of developing postpartum infections.

Interestingly, epinephrine may increase the secretion of ROS in puerperas’ neutrophils under either alpha-AR or beta-AR/beta-1-AR/beta-3-AR desensitization. A recent study suggests that neutrophil functional activity increases via alpha-1-AR (Kanashiro *et al*., 2019); therefore, it can be assumed that this AR subtype is in abundance in the neutrophil membrane of puerperas and provides increased level of ROS in the blood.

## CONCLUSION

We can conclude the latex-induced oxidative burst of female blood neutrophils correlates with the stage of the reproductive process: it decreases during pregnancy, reaching the lowest value during childbirth, and increases significantly in the first hours after childbirth. This is probably linked to progesterone levels and its ability to change the adrenergic receptor ratio in the cell membranes via genomic pathways. It is necessary for the best possible uterine contractile activity and the development of maternal immunological tolerance to the semiallogeneic embryo. In the first day after childbirth, neutrophil alpha-1-adrenergic receptors become highly active. They activate neutrophil NADPH-oxidase and, thus, cause ROS generation; the latter act as protective factors. However, alphaor beta-adrenergic agonists inhibit neutrophil oxidative activity. Our results give every reason to believe that, if physiologically necessary, a mechanism is formed to reduce the excess activity of neutrophils after childbirth. And stressful conditions in the postpartum period can suppress the ability of neutrophils to release ROS, which increases the risk of postpartum infections.

**Contributions:** All authors contributed to the conception and design of this study and are accountable for all aspects of the study. Oksana Olegovna Zaitseva, Marta Igorevna Sergushkina, and Olga Nurzadinovna Solomina - data collection and analysis, and interpretation of data; Tatiana Vitalievna Polezhaeva, Oksana Olegovna Zaitseva, Inna Gennadievna Paturova, Victor Ivanovich Tsirkin - writing the article, analysis and interpretation of the study data. Tatiana Vitalievna Polezhaeva, Oksana Olegovna Zaitseva, Andrey Nikolaevich Khudyakov, Svetlana Leonidovna Dmitrieva - drafting of the article; all authors revised it for important intellectual content, and read and approved the final article to be published.
